# Quantitative proteomic analysis of intracerebral hemorrhage in rats with a focus on brain energy metabolism

**DOI:** 10.1002/brb3.1130

**Published:** 2018-10-11

**Authors:** Tao Liu, Jing Zhou, Hanjin Cui, Pengfei Li, Haigang Li, Yang Wang, Tao Tang

**Affiliations:** ^1^ Institute of Integrative Medicine Xiangya Hospital, Central South University Changsha China; ^2^ Department of Gerontology Traditional Chinese Medicine Hospital Affiliated to Xinjiang Medical University Urumqi China; ^3^ Department of Pharmacy Changsha Medical University Changsha China

**Keywords:** animal models, energy metabolism, intracerebral hemorrhage, iTRAQ, Proteomics

## Abstract

**Introduction:**

Intracerebral hemorrhage (ICH) is a lethal cerebrovascular disorder with a high mortality and morbidity. The pathophysiological mechanisms underlying ICH‐induced secondary injury remain unclear.

**Methods:**

To examine one of the gaps in the knowledge about ICH pathological mechanisms, isobaric tag for relative and absolute quantification (iTRAQ)‐based liquid chromatography‐tandem mass spectrometry (LC‐MS/MS) was used in collagenase‐induced ICH rats on the 2nd day.

**Results:**

A total of 6,456 proteins were identified with a 1% false discovery rate (FDR). Of these proteins, 126 and 75 differentially expressed proteins (DEPs) were substantially increased and decreased, respectively. Based on Gene Ontology (GO), Kyoto Encyclopedia of Genes and Genomes (KEGG), and STRING analyses, the protein changes in cerebral hemorrhage were comprehensively evaluated, and the energy metabolism in ICH was anchored. The core position of the nitrogen metabolism pathway in brain metabolism in ICH was found for the first time. Carbonic anhydrase 1 (Ca1), carbonic anhydrase 2 (Ca2), and glutamine synthetase (Glul) participated in this pathway. We constructed the protein–protein interaction (PPI) networks for the energy metabolism of ICH, including the Atp6v1a‐Atp6v0c‐Atp6v0d1‐Ppa2‐Atp6ap2 network.

**Conclusions:**

It seems that dysregulation of energy metabolism, especially nitrogen metabolism, may be a major cause in secondary ICH injury. This information provides novel insights into secondary events following ICH.

## 1 INTRODUCTION

Intracerebral hemorrhage (ICH) is a lethal form of cerebrovascular disorder with high mortality and morbidity (Hemphill et al., 2015; Lan, Han, Li, Li, et al., 2017; Lan, Han, Li, Yang, & Wang, 2017). Considering the aging of populations, the incidence of ICH is expected to increase (van Asch et al., 2010). Currently, there is no effective therapy available to improve the clinical end points of ICH, because of the combined effect of primary injury and secondary damage (Fiorella, Zuckerman, Khan, Ganesh Kumar, & Mocco, 2015; Hemphill et al., 2015; Mittal & LacKamp, 2016). The primary injury is mainly caused by the mechanical oppression and destruction to the surrounding tissue by hematoma formation (Wang, 2010; Zheng, Chen, Zhang, & Hu, 2016). The secondary injury, for the most part, results from the presence of intraparenchymal blood that may be dependent upon the initial hematoma volume or age (Wang, 2010), and it may occur via many parallel pathological pathways (Wu et al., 2010). Most studies of the secondary brain injury focused on excitotoxicity, coagulation cascade, oxidative stress (OS), inflammation, iron toxicity (Li, Han, Lan, Gao, et al., 2017; Li, Han, Lan, Hong, et al., 2017; Li, Wan, Lan, et al., 2017; Wu, Wu, Xu, Wang, & Wang, 2011), ferroptosis, necrosis, and autophagy (Hu et al., 2016; Lan, Han, Li, Li, et al., 2017; Lan, Han, Li, Yang, et al., 2017; Li, Han, Lan, Gao, et al., 2017; Li, Han, Lan, Hong, et al., 2017; Li, Wan, Lan, et al., 2017; Wang & Tsirka, 2005; Zhang et al., 2017). However, the precise pathophysiological mechanisms underlying ICH‐induced secondary injury remain far from being completely elucidated.

Recent advances in proteomics technologies offer opportunities to study the global protein landscape and identify the new pathophysiological aspect. Many proteomic studies on experimental ischemic stroke have been reported, whereas there were only two proteomic studies in rat models of hemorrhagic stroke. Furthermore, these two studies have some limitations: One of the studies employed a traditional 2D gel combined with mass spectrometry (MS) (Chiu et al., 2012). This approach still suffers from low recognition sensitivity and linearity and a relatively low‐throughput, and it cannot analyze highly basic/hydrophobic proteins (Aggarwal, Choe, & Lee, 2006; Sethi, Chourasia, & Parhar, 2015). Moreover, the major disadvantage of two‐dimensional gel electrophoresis (2‐DE) is poor reproducibility or gel to gel variability (Sethi et al., 2015). Another study utilized a label‐free liquid chromatography‐tandem mass spectrometry (LC‐MS/MS)‐based relative quantification approach to evaluate rat brains with ICH (Ren et al., 2014). Unfortunately, the label‐free method identifies fewer proteins with accurate quantification, especially those of lower abundance (Craft, Chen, & Nairn, 2013). More importantly, although the study identified more than 600 proteins, most of them were already reported in known research. It is necessary to use advanced proteomic methods to identify some novel proteins and elucidate the pathophysiological mechanisms of ICH. With the development of proteomic techniques, isobaric tag for relative and absolute quantitation (iTRAQ) followed by multidimensional liquid chromatography and tandem MS provides a more powerful methodology for quantitative proteomics (Wang et al., 2013). Thus, to explore the potential mechanisms of ICH, we used iTRAQ‐based quantitative proteomics in this study.

To the best of our knowledge, iTRAQ‐based quantitative proteomics has not been used to study ICH. To gain a better understanding of ICH‐induced secondary injury, we sought to apply iTRAQ‐based LC‐MS/MS analysis. In this study, we established a protein sketch of ICH‐induced secondary brain injury by comparing the proteomes of the sham‐operated and ICH‐operated groups. Based on the bioinformatic analysis, we aimed to reveal the novel processes involved in ICH. In addition, major related proteins were further validated by western blotting.

## 2 MATERIALS AND METHODS

### 2.1 Animal experiments

Sprague‐Dawley (*SD*) male rats (6–7 weeks, 180–200 g), specific pathogen‐free (SPF) grade, were provided by the Laboratory Animal Centre of Central South University (CSU). They were housed three per cage and received food and water ad libitum under controlled environmental conditions (room temperature 21–25°C, room relative humidity 40%–60%, 12‐hr day–night cycle with lights) for five days to adapt to the environment. The study was verified and approved by the Animal Ethics Committee of CSU.

### 2.2 Experimental design and statistical rationale

For iTRAQ‐based LC‐MS/MS analysis, collagenase‐induced ICH and sham‐operated rats brain samples were analyzed in one technical replicate. Forty rats were randomly (Li, Han, Lan, Gao, et al., 2017; Li, Han, Lan, Hong, et al., 2017; Li, Wan, Lan, et al., 2017) divided into two groups: (a) Sham‐operated group (*n* = 20): Rats underwent the ICH procedure without collagenase infusion; and (b) ICH group (*n* = 20): Rats underwent the ICH procedure with collagenase infusion. All rats were evaluated using the modified neurological severity score (mNSS) at 24 and 48 hr. At 48 hr after ICH, the rats were deeply anesthetized with 3% pentobarbital sodium (50 mg/kg). Rats were transcardially perfused with 0.9% ice‐cold saline and then sacrificed by decapitation. Equal amounts of protein from each of the four brain tissue samples were mixed to generate one normalization pool, and the proteomic study was performed. The results of this study are presented as the means ± *SD*. Statistical comparisons between means were assessed by two‐tailed Student's *t* test using SPSS software (IBM, v19). At least a 1.3‐fold change and a *p*‐value <0.05 were considered as significant.

### 2.3 Induction of ICH

A collagenase‐induced ICH model was established according to previous reports (Peng, Yang, & Yang, 2014; Wang et al., 2016; Zhou et al., 2010). All rats were anesthetized with 3% pentobarbital sodium (50 mg/kg) through an intraperitoneal injection and then fixed on a stereotaxic frame (Stoelting Co., USA) in the prone position. Following a midline scalp incision, a small burr hole was drilled 3.2 mm to the right and 1.4 mm posterior of the bregma. A 5‐μl Hamilton syringe was lowered into the right globus pallidus (5.6 mm ventral to cortical surface), and 0.5 U of collagenase (Sigma Co., USA; Type VII) in 2.5 μl of 0.9% sterile saline was slowly injected over 2 min, with the needle remaining in place for an additional 5 min. After the hole was sealed and the scalp was sutured, the rats were housed individually in a warm cage to recover. For the sham‐operated group, the animals were administered 2.5 μl of 0.9% sterile saline instead of collagenase at the same site.

### 2.4 Pre‐proteomic assessment

Neurological Evaluation—All rats were evaluated using the mNSS (Xing et al., 2016; Zhou et al., 2017) at 24 and 48 hr. Neurological function was graded on a scale of 0–18 (normal score 0; maximal deficit score 18). The mNSS is a composite of motor, sensory, reflex, and balance tests. In the severity scores of injuries, 13–18 points indicate severe injury; 7–12 points indicate moderate injury; and 1–6 points indicate mild injury.

Hematoxylin–eosin (H&E) staining—At 48 hr after ICH, the rats were sacrificed, and then, the part of hemorrhagic hemisphere was fixed in 10% neutral‐buffered formalin. The tissue was embedded in paraffin, and sections were H&E stained.

### 2.5 Tissue preparation and protein extraction

The whole right or hemorrhagic hemisphere excluding the cerebellum and olfactory lobe (hereafter called the ipsilateral hemisphere) was selected in our proteomic experiment. To extract proteins, the ipsilateral hemispheres were ground into powder in liquid nitrogen, and proteins were then extracted with ice‐cold lysis buffer (7 M urea, 4% CHAPS, 30 mM HEPES) containing 1 mM PMSF and 2 mM EDTA (final concentration). After five minutes, 10 mM DTT (final concentration) was added to the samples. The suspension was sonicated at 200 Watts for 15 min and then centrifuged at 4°C and 30,000 × g for 15 min. The supernatant was mixed well with a fivefold excess of 10% TCA chilled acetone and incubated at −20°C overnight. After centrifugation at 4°C, 30,000 × g for 15 min, the supernatant was discarded. The precipitate was washed with chilled acetone three times. The pellet was air‐dried and dissolved in lysis buffer. The suspension was sonicated at 200 W for 15 min and centrifuged at 4°C, 30,000 × g for 15 min. To reduce the disulfide bond of the supernatant, 10 mM DTT was added at 56°C for 1 hr. Subsequently, 55 mM IAM was added to block the cysteines, and the samples were incubated in a dark room for 45 min. The supernatant was mixed well with a fivefold excess of precooled acetone for 2 hr at −20°C to precipitate proteins. After centrifugation at 4°C, 30,000 × *g*, the supernatant was discarded, and the pellet was air‐dried for 5 min, dissolved in 500 μl of 0.5 M lysis buffer, and sonicated at 200 W for 15 min. Finally, samples were centrifuged at 4°C, 30 000 g for 15 min. The supernatant was transferred and kept in aliquots at −80°C (longer term) or at −20°C (shorter duration). Protein concentration was done using a BCA quantification kit (Sigma Co., USA).

### 2.6 iTRAQ labeling and strong cation exchange (SCX) fractionation

The protein digestion process followed the filter‐aided sample preparation (FASP) (Sun & Jiang, 2013; Wisniewski, Zougman, & Mann, 2009; Wisniewski, Zougman, Nagaraj, & Mann, 2009). Briefly, each sample of 200 μg of protein was reduced, alkylated, and then digested with trypsin (Promega Co., USA) overnight at 37°C. The following day, the samples were centrifuged at 12,000 g for 20 min. Subsequently, 50 μl of 1 M TEAB was added, and the samples were centrifuged for 20 min under the same conditions. The final peptides were transferred to a new tube. Afterward, the peptides were processed according to the manufacturer's instructions for 8‐plex iTRAQ reagent (Applied Biosystems). Samples were labeled with the iTRAQ tags as follow: Sample ICH‐operated group (tag 117) or Sample sham group (tag 118). The peptides were labeled with the isobaric tags and incubated at room temperature for 2 hr. All labeled peptide mixtures were then pooled and evaporated to dryness by vacuum centrifugation.

SCX chromatography was performed with a LC‐20AD HPLC Pump system (Shimadzu, Japan). The iTRAQ‐labeled tryptic peptides were reconstituted with buffer A (20 mM HCOONH4, pH 10) and loaded onto a 2 × 150 mm Gemini‐NX C18 SCX column containing 3‐μm particles (Phenomenex) with 80% acetonitrile 20 mM HCOONH4 as buffer B. The peptides were eluted at a flow rate of 200 μl/min with a gradient of different concentrations of buffer B for 1 hr. Elution was monitored by measuring the absorbance at 214 nm/280 nm, and fractions were collected every 1 min (Wang et al., 2011). The eluted peptides were pooled into 24 fractions, which were vacuum‐dried and acidized in 50% TFA for further nanoLC‐MS/MS analysis (Hua et al., 2016).

### 2.7 NanoLC–MS/MS analysis

The peptides were dissolved in a sample solution (0.1% FA, 2% ACN) and then centrifuged at 4°C, 13,500 g for 20 min. The nanoLC–MS/MS was performed using a Q Exactive MS (Thermo Scientific) interfaced with a Thermo Dionex Ultimate 3,000 RSLC nano system. The supernatant was separated from the PepMap C18 RP column (2 μm, 75 μm × 150 mm, 100 A) at a flow rate of 300 nl/min. Peptides were eluted from the HPLC column by using a binary linear gradient of 4%–90% mobile phase B (80% ACN, 0.1% FA) for 65 min. The eluted peptides were directly detected by Q Exactive online. A data‐dependent top 20 method was selected for MS data acquisition, and the most abundant precursor ions from the survey scan (350 to 1,800 m/z) were chosen for HCD (high‐energy collisional dissociation) fragmentation. Automatic gain control (AGC) was utilized to determine the target value. Survey scans were acquired at a resolution of 70,000 at *m*/*z* 200, and the resolution for HCD spectra was set to 17,500 at *m*/*z* 200 with an isolation width of 2 *m*/*z* (Yu et al., 2017). The underfill ratio, which specifies the minimum percentage of the target value likely to be reached at the maximum‐fill time, was defined as 0.1%. The peptide recognition mode was adopted throughout the process.

### 2.8 Analysis of proteomics data

#### 2.8.1 Data analysis

Raw data files acquired from the Thermo Q Exactive™ LC‐MS/MS System were converted into peak lists (.mgf) by Proteome Discoverer™ Software (version 1.4, Thermo Fisher Scientific, Waltham, MA). MGF peak files as the input file (ProteinPilot™ Software version 5.0.1; AB Sciex, Foster, CA, USA) were used to conduct deep protein quantitation and identification with a rat proteome database (updated on 07/07/2016, https://www.uniprot.org/proteomes/UP000002494) that contains 26,479 reviewed entries. Protein pilot search parameters were set as follows: Sample Type (iTRAQ 8plex), Cys Alkylation (MMTS), Digestion (Trypsin), Database (RAT.fasta), ID Focus (biological modifications), Search Effort (thorough ID), [Unused ProtScore (Conf)] (>0.05), FDR Analysis (Yes), and User Modified Parameter Files (No). Thus, there were no prior settings for the search with the number of missed and/or nonspecific cleavages, no prior fixed/variable modifications, and no prior mass tolerance for fragment ions. In addition, the identified peptides were filtered by false discovery rate (FDR) 1% based on the decoy automatic database searching method. All MS data have been deposited in the PRIDE Archive with the dataset identifiers PXD006437. A strict set of criteria was imposed on the quantitative data analysis. Briefly, identified proteins must contain at least two unique high‐scoring peptides (peptide confidence >95%); quantitative proteins must be observed across two technical replicates; and identified proteins (an average ratio change >1.3 or <0.77) were considered differentially expressed proteins.

#### 2.8.2 Bioinformatics analysis

Gene Ontology (GO) is an international standardization of a gene function classification system. GO analysis of differentially expressed proteins (DEPs) was performed with the QuickGO Database (https://www.ebi.ac.uk/QuickGO/). With DEPs’ UniProt accession searching against QuickGO Database, categorical annotation was supplied as GO biological process (BP), molecular function (MF), and cellular component (CC).

The Kyoto Encyclopedia of Genes and Genomes (KEGG) Pathway database (https://www.genome.jp/kegg/pathway.html) is a collection of manually drawn graphical diagrams, called KEGG pathway maps, representing molecular pathways for metabolism, genetic information processing, environmental information processing, cellular processes, human diseases, and drug development. To find the experimental impact on rats, DEPs’ UniProt accessions were converted to KEGG accession and then mapped to the KEGG rat pathway.

STRING 10.0 (https://www.string-db.org/) was used to explore protein–protein interacting networks and functional relations in DEPs.

### 2.9 Western blotting

The protein contents of the supernatants were quantified using a BCA quantification kit (Sigma Co., USA). Proteins (50 μg) from the supernatant of each sample were separated by SDS‐PAGE and transferred to a PVDF membrane, which was subsequently blocked with 5% skim milk in TBST buffer containing 20 mM Tris–HCl, 150 mM NaCl, and 0.05% Tween 20 (pH 7.5) for 2 hr at room temperature. The membranes were incubated with the following primary antibodies rabbit anti‐Ca2 (1:1,000, Abcam), rabbit anti‐Atp6vla (1:2,000, Abcam), mouse anti‐β‐actin (1:5,000, Proteintech) overnight and then incubated with horseradish peroxidase‐labeled anti‐rabbit secondary antibody (1:6,000, Proteintech) and anti‐mouse secondary antibody (1:5,000, Proteintech) for 2 hr at room temperature. Bands were visualized using the Bio‐Rad ChemiDoc XRS digital documentation system (Bio‐Rad, Hercules, CA, USA) and quantified using ImageJ software. The amount of protein expression is presented relative to the levels of β‐actin.

## 3 RESULTS

### 3.1 Neurological deficits and H&E staining

**Figure 1:**
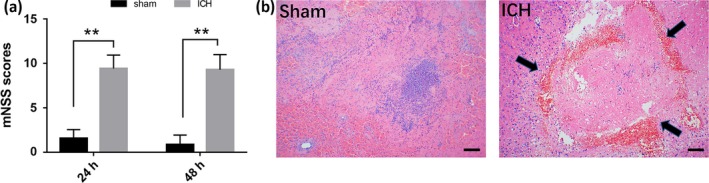
(a) The mNSS values for the two groups at 24 and 48 hr post‐ICH. ICH caused significant neurological impairment compared with the sham group. Error bars indicate *SD*; **p < 0.01 versus sham at 24 and 48 hr; *n* = 20 per group. (b) Representative H&E staining of brain tissue shows the hemorrhagic region and morphological changes. A typical ICH‐induced annular hemorrhage and edema at day 2, compared with the sham group. Original magnification, ×100. Scale bar = 100 μm. *n* = 5 per group

At the two‐time points (24 and 48 hr), we performed mNSS tests on the sham and ICH groups. In the sham group, the mNSS scores were low at all time points. Conversely, the mNSS scores in the ICH group remained higher than those of the sham group (shown in Figure 1a). H&E staining was used to evaluate the histopathological changes at 48 hr after ICH. Microscopic examination of the H&E‐stained brain tissue showed a typical ICH‐induced annular hemorrhage and edema at day 2 (shown in Figure 1b).

### 3.2 Identification of DEPs between the sham and ICH groups

We identified 6,456 proteins with an FDR of less than 1% and unique peptide matches ≥1. Of these identified proteins, 4,578 were repeatedly identified in two replicates. A total of 201 significantly altered protein hits (75 downregulated and 126 upregulated) were found responsive to ICH using the criteria as described above. Details on the DEPs are listed in Supporting Information Table S1.

### 3.3 Bioinformatic analysis of the DEPs

**Figure 2:**
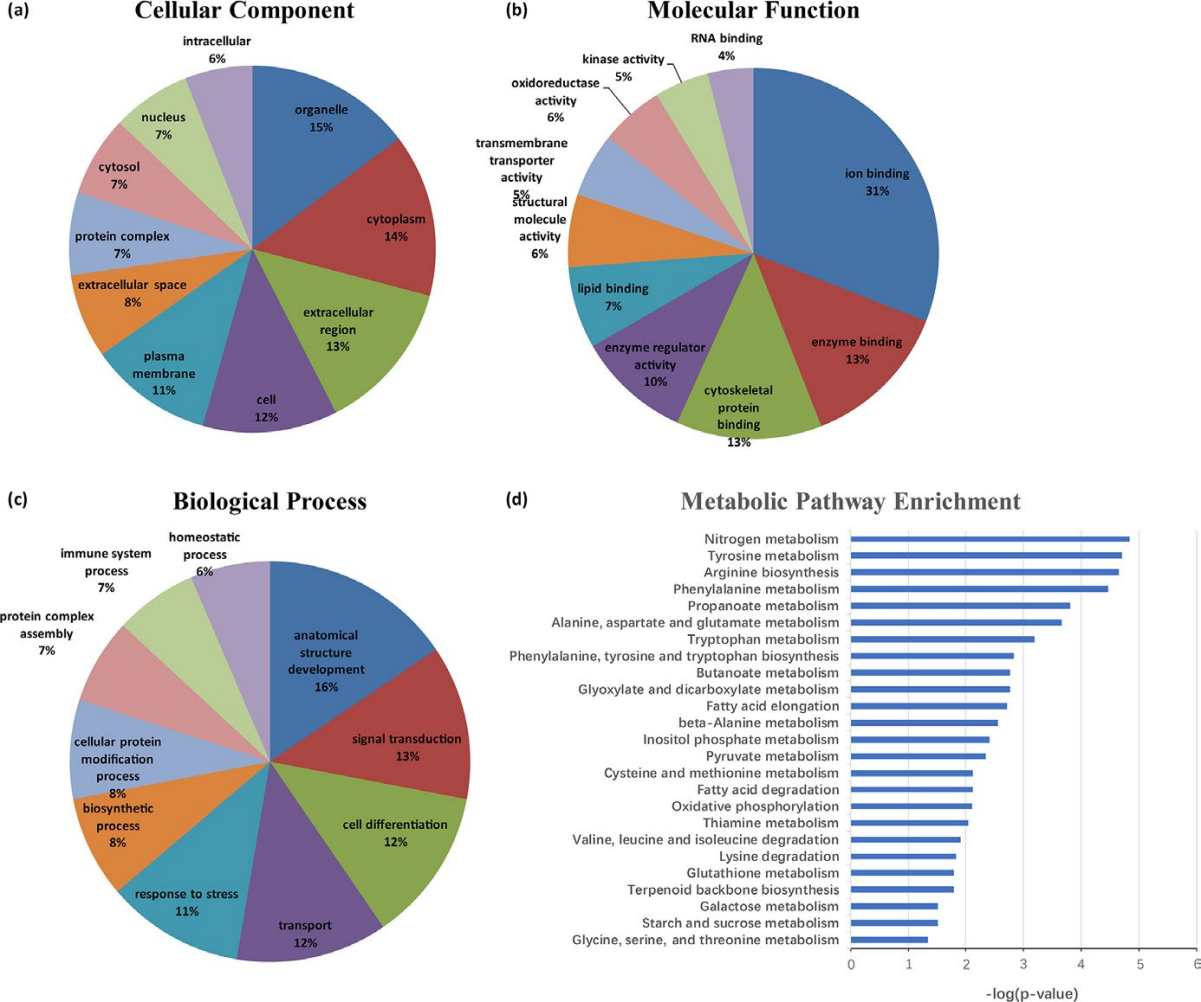
GO analysis of the differentially expressed proteins (DEPs). The 201 DEPs were imported into QuickGO software for GO annotation according to their cellular component (a), molecular function (b), and biological process (c). (d) The Metabolic pathway enrichment analysis was conducted, and the significantly enriched categories were recorded. 25 Metabolic pathways were significantly perturbed with *p* < 0.05, which corresponds to −log(*p*‐value) > 1.3 identified by the method. Nitrogen metabolism was found to be the most significant (−log(*p*‐value) = 4.84)

Two hundred and one DEPs were imported into QuickGO software for GO annotation. In the cellular component of GO analysis, the highest proportion of DEPs was located in the organelle (15%). Proteins located in the cytoplasm (14%) and the extracellular region (13%) were the next two largest groups in the cellular component category (Figure 2a), while ion binding (31%), enzyme binding (13%), and cytoskeletal protein binding (13%) were the major functions under the category of molecular functions (Figure 2b). In addition, the GO analysis of biological process found that 16% of the DEPs were related to anatomical structure development, followed by signal transduction (13%), cell differentiation (12%), transport (12%), and response to stress (11%) (Figure 2c).

Based on 201 DEPs, the KEGG pathways analysis identified 186 canonical pathways (Supporting Information Table S2). In these pathways, we found that most of the proteins were enriched in the Metabolic pathways, followed by the Complement and coagulation cascades, Phagosome, cAMP signaling and Calcium signaling pathway (Table 1). The Metabolic pathways were further analyzed using the enrichment analysis method. Twenty‐five Metabolic pathways were significantly perturbed at *p* < 0.05, which corresponds to −log(*p*‐value) > 1.3 identified by the method (Figure 2d). Nitrogen metabolism was the most significant (−log(*p*‐value) = 4.84), and three proteins (carbonic anhydrase 1 (Car1), carbonic anhydrase 2 (Ca2), and glutamine synthetase (Glul) were involved in this pathway.

**Table 1:** Pathway enrichment analysis of differential expression of proteins

Pathway ID	Pathway name	Proteins quantity
rno01100	Metabolic pathways	18
rno04610	Complement and coagulation cascades	8
rno04145	Phagosome	7
rno04024	cAMP signaling pathway	7
rno04020	Calcium signaling pathway	7
rno04728	Dopaminergic synapse	6
rno04070	Phosphatidylinositol signaling system	6
rno04721	Synaptic vesicle cycle	5
rno04722	Neurotrophin signaling pathway	5
rno04750	Inflammatory mediator regulation of TRP channels	4

**Figure 3:**
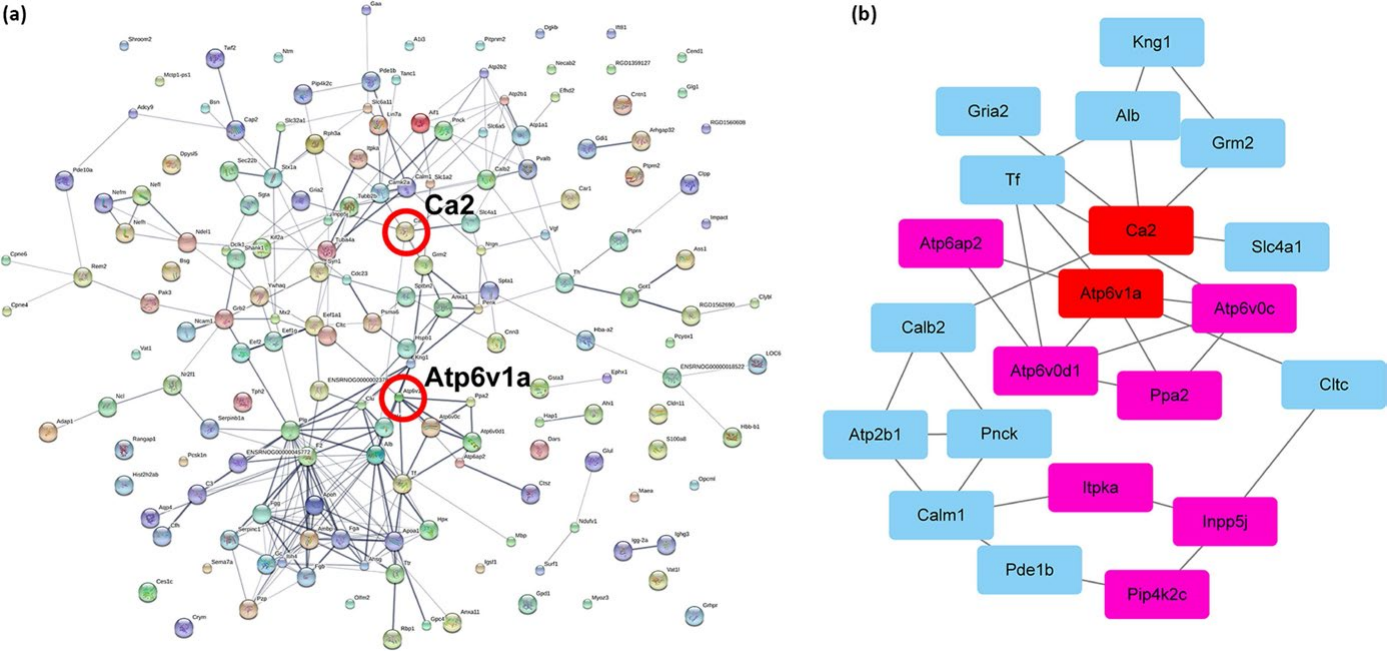
(a) Protein–protein interacting networks and functional relations in DEPs were analyzed with STRING 10.0. Of the 201 DEPs, 128 proteins (86 upregulated and 42 downregulated) were obtained, which were associated with at least one other protein. In the network, the proteins are represented as nodes. The line thickness indicates the strength of data support. Ca2 and Atp6v1a were selected for validation. (b) Protein–protein interactions (PPI) were constituted by using the Cytoscape software based on STRING analysis

To determine the relationship between differentially expressed proteins, we searched the STRING database for the functional relations and networks of DEPs. When the 201 DEPs were input, we extracted a network of 165 genes from the STRING database using default settings (Figure 3a). Finally, 128 proteins (86 upregulated and 42 downregulated) were obtained that were associated with at least one other protein. Among the interacting proteins, coagulation cascade‐related proteins, energy metabolism‐related proteins, and inflammation‐related proteins had a high level of coexpression. In the STRING analysis, some proteins that showed significant results in terms of the combined score interact with each other, such as Fga, Fgg, F2, Atp6v0d1, Atp6v1a Atp6v0c, C3, and Cfh (Supporting Information Table S3).

**Figure 4:**
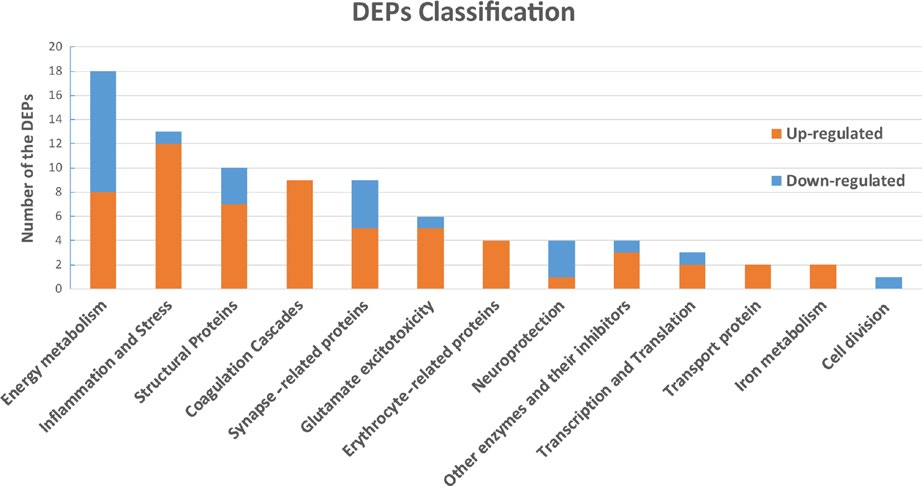
Of the 201 DEPs, we focused on 85 significant DEPs which were selected based on GO, KEGG, STRING analysis. These DEPs were classified into 13 categories based on stroke pathophysiology. Upregulated and downregulated DEPs are shown by orange and blue bars, respectively

In this study, to improve specificity and accuracy, we focused on 85 significant DEPs that were selected based on GO, KEGG, and STRING analyses (Table 2). Subsequently, the proteins were classified into 13 categories based on stroke pathophysiology: Energy Metabolism, Inflammation and Stress, Structural Proteins, Coagulation Cascades, Synapse‐related proteins, Glutamate excitotoxicity, Erythrocyte related proteins, Neuroprotection, Other enzymes and their inhibitors, Transcription and Translation, Transport protein, Iron metabolism, and Cell division. As shown in Figure 4, Energy metabolism, Inflammation and Stress, Structural Proteins, Coagulation Cascade, and Synapse‐related proteins ranked as the top five. In addition, the proportions of downregulated proteins in Energy metabolism were large, while the upregulated proteins were mostly related to Inflammation and Stress, Structural Proteins, and Coagulation Cascade.

**Table 2:** Quantitative information of the selected regulated proteins in collagenase‐induced ICH rats

Accession	Unused^a^	%Cov (95)^a^	Peptides (95%)^a^	Gene	Protein description	ICH/Sham^b^
Energy metabolism						
B0BNN3	8.74	21.84	6	Car1	Carbonic anhydrase 1	5.95
Q6AXS4	9.02	16	8	Atp6ap2	Renin receptor	2.54
P06685	50.25	51.81	209	Atp1a1	Sodium/potassium‐transporting ATPase subunit alpha‐1	2.43
Q5M7T6	24.36	50.14	19	Atp6v0d1	ATPase, H + transporting, lysosomal 38 kDa, V0 subunit d1	2.17
P63081	13.12	49.03	16	Atp6v0c	V‐type proton ATPase 16 kDa proteolipid subunit	1.92
G3V9W5	14.54	28.50	10	Pip4k2c	Phosphatidylinositol 5‐phosphate 4‐kinase type‐2 gamma	1.43
P17105	24.55	36.82	14	Itpka	Inositol‐trisphosphate 3‐kinase A	1.34
P27139	25.31	55.38	26	Ca2	Carbonic anhydrase 2	1.32
D4A133	65.3	69.21	87	Atp6v1a	Protein Atp6v1a	0.55
P09034	13.4	23.30	9	Ass1	Argininosuccinate synthase	0.59
P04177	10.48	12.45	7	Th	Tyrosine 3‐monooxygenase	0.65
B5DEN4	34.63	64.46	37	RGD1562690	L‐lactate dehydrogenase	0.66
Q5XIH3	35.39	58.84	31	Ndufv1	NADH dehydrogenase (Ubiquinone) flavoprotein 1	0.67
D4A830	12.09	27.63	8	Ppa2	Pyrophosphatase (inorganic) 2	0.70
Q6P7A9	19.16	19.41	14	Gaa	Lysosomal alpha‐glucosidase	0.70
Q9JMC1	5.37	7.79	6	Inpp5j	Phosphatidylinositol 4,5‐bisphosphate 5‐phosphatase A	0.72
P13221	44.91	71.43	54	Got1	Aspartate aminotransferase, cytoplasmic	0.74
P14604	24.5	53.45	20	Echs1	Enoyl‐CoA hydratase, mitochondrial	0.76
Inflammation and stress						
Q63041	68.56	29.87	53	Pzp	Alpha‐1‐macroglobulin	25.40
M0RBF1	71.38	31.21	49	C3	Complement C3	23.34
P24090	12.52	35.80	10	Ahsg	Alpha‐2‐HS‐glycoprotein	8.89
P02770	88.15	70.72	91	Alb	Serum albumin	6.54
P04639	10.01	29.34	8	Apoa1	Apolipoprotein A‐I	6.04
G3V913	4.48	21.84	3	Hspb1	Heat‐shock 27 kDa protein 1	5.89
F1M983	23.12	13.29	15	Cfh	Protein Cfh	4.80
P20760	14	30.43	8	Igg‐2a	Ig gamma‐2A chain C region	3.17
P07150	10.1	22.83	11	Anxa1	Annexin A1	1.85
Q4G075	9.62	20.05	6	Serpinb1a	Leukocyte elastase inhibitor A	1.85
P20762	3.3	8.82	3	Ighg3	Ig gamma‐2C chain C region	1.52
P60901	16.58	37.40	10	Psma6	Proteasome subunit alpha type‐6	0.55
Structural proteins						
F1LRZ7	54.4	41.26	55	Nefh	Neurofilament heavy polypeptide	1.61
Q5XIF6	6.82	84.38	331	Tuba4a	Tubulin alpha‐4A chain	1.60
P12839	79.88	49.88	86	Nefm	Neurofilament medium polypeptide	1.57
P19527	55.56	58.49	87	Nefl	Neurofilament light polypeptide	1.49
P52481	26.57	36.06	18	Cap2	Adenylyl cyclase‐associated protein 2	1.39
P62994	15.5	41.01	10	Grb2	Growth factor receptor‐bound protein 2	1.36
F1MAK3	32.05	9.42	18	Arhgap32	Protein Arhgap32	1.34
Q78PB6	13.54	32.17	9	Ndel1	Nuclear distribution protein nudE‐like 1	0.61
Q3KRE8	2	78.43	272	Tubb2b	Tubulin beta‐2B chain	0.66
F1M9F9	1.55	3.44	4	Ahi1	Jouberin	0.75
Coagulation cascades						
Q7TQ70	28.13	27.88	24	Fga	Ac1873	10.40
P08932	30.07	40.93	17	Kng1	T‐kininogen 2	7.59
P02680	18.4	29.44	16	Fgg	Fibrinogen gamma chain	6.10
Q5I0M1	8.52	13.04	4	Apoh	Apolipoprotein H	4.22
P14480	20.38	41.75	4	Fgb	Fibrinogen beta chain	3.94
Q01177	13.7	10.47	7	Plg	Plasminogen	3.42
Q5M7T5	11.92	15.27	7	Serpinc1	Protein Serpinc1	2.93
P06765	2	7.62	2	Pf4	Platelet factor 4	2.27
G3V843	8.19	8.75	8	F2	Prothrombin	2.16
Synapse‐related proteins						
P11275	35.04	64.44	76	Camk2a	Calcium/calmodulin‐dependent protein kinase type II subunit alpha	2.82
P09951	91.64	72.30	152	Syn1	Synapsin‐1	2.41
Q9WV48	48.55	16.94	35	Shank1	SH3 and multiple ankyrin repeat domains protein 1	1.77
P32851	17.31	46.53	23	Stx1a	Syntaxin‐1A	1.51
P05197	67.96	45.92	46	Eef2	Elongation factor 2	1.47
F1LQG0	10	9.70	7	Hap1	Huntingtin‐associated protein 1	0.55
Q642B0	12.3	14.00	8	Gpc4	Glypican 4	0.55
O35458	22.02	25.33	11	Slc32a1	Vesicular inhibitory amino acid transporter	0.68
O70150	2	7.00	3	Pnck	Calcium/calmodulin‐dependent protein kinase type 1B	0.74
Glutamate excitotoxicity						
G3V6R0	1.6	34.81	49	Slc1a2	Excitatory amino acid transporter 2	14.11
P19491	42.7	31.48	38	Gria2	Glutamate receptor 2	1.82
P31421	12.34	9.40	9	Grm2	Metabotropic glutamate receptor 2	1.70
P09606	34.86	55.50	57	Glul	Glutamine synthetase	1.51
F1M779	171.28	58.69	213	Cltc	Clathrin heavy chain	1.49
O70593	16.61	34.08	12	Sgta	Small glutamine‐rich tetratricopeptide repeat‐containing protein alpha	0.68
Erythrocyte‐related proteins						
B1H216	22.63	76.76	49	Hba‐a2	Hemoglobin alpha, adult chain 2	67.04
Q62669	2	85.71	47	LOC103694855	Protein Hbb‐b1	14.25
P11517	6.07	97.28	70	LOC689064	Hemoglobin subunit beta‐2	12.50
D4A678	18.61	9.52	27	Spta1	Protein Spta1	3.80
Neuroprotection						
P62161	34.4	89.93	80	Calm1	Calmodulin	2.00
F1LP80	25.08	23.99	14	Vgf	Neurosecretory protein VGF	0.37
P04094	4.1	11.15	3	Penk	Proenkephalin‐A	0.47
F1LNY3	83.63	54.51	82	Ncam1	Neural cell adhesion molecule 1	0.50
Other enzymes and their inhibitors						
Q5EBC0	24.09	17.47	15	Itih4	Interalpha‐trypsin inhibitor, heavy chain 4	5.57
Q64240	2.92	8.02	3	Ambp	Protein AMBP	1.92
Q9R1T3	5.06	12.75	4	Ctsz	Cathepsin Z	1.50
Q01066	22.99	25.61	16	Pde1b	Calcium/calmodulin‐dependent 3′,5′‐cyclic nucleotide phosphodiesterase 1B	0.63
Transcription and translation						
P62630	43.71	71.00	50	Eef1a1	Elongation factor 1‐alpha 1	1.83
Q68FR6	22.83	36.61	17	Eef1g	Elongation factor 1‐gamma	1.43
P50398	26.08	54.36	61	Gdi1	Rab GDP dissociation inhibitor alpha	0.46
Transport protein						
P23562	28.19	20.39	19	Slc4a1	Band 3 anion transport protein	15.19
Q4KM74	13.96	43.26	8	Sec22b	Vesicle‐trafficking protein SEC22b	1.39
Iron metabolism						
P20059	20.7	34.78	17	Hpx	Hemopexin	3.11
P12346	2	48.28	41	Tf	Serotransferrin	2.62
Cell division						
F1LRQ6	2	3.15	3	Cdc23	CDC23 (Cell division cycle 23, yeast, homolog), isoform CRA_b	0.69

Note

Unused (ProtScore): A measure of the protein confidence for a detected protein, calculated from the peptide confidence for peptides from spectra that are not already completely “used” by higher scoring winning proteins.

% Cov (95): The percentage of matching amino acids from identified peptides having confidence greater than or equal to 95% divided by the total number of amino acids in the sequence.

Peptides (95%): The number of distinct peptides having at least 95% confidence.

a These data are derived from RUN 1.

b The relative quantitative data that repeated once from two groups were averaged and used in the computations. The DEPs were classified according to their participation in the key molecular events of ICH pathophysiology.

Based on the above bioinformatics analysis, energy metabolism was selected for further study. Cytoscape software was used for protein–protein interaction (PPI) analysis of the DEPs related to energy metabolism (Figure 3b). In the PPI network, Ca2 was in the core position. In addition, we found that ATPase, H^+^ transporting, Lysosomal 38 kDa, V0 subunit d1 (Atp6v0d1), Protein Atp6v1a (Atp6v1a), V‐type proton ATPase 16 kDa proteolipid subunit (Atp6v0c), Renin receptor (Atp6ap2), and Pyrophosphatase (inorganic) 2 (Ppa2) constituted a complete network.

### 3.4 Validation of DEPs

**Figure 5:**
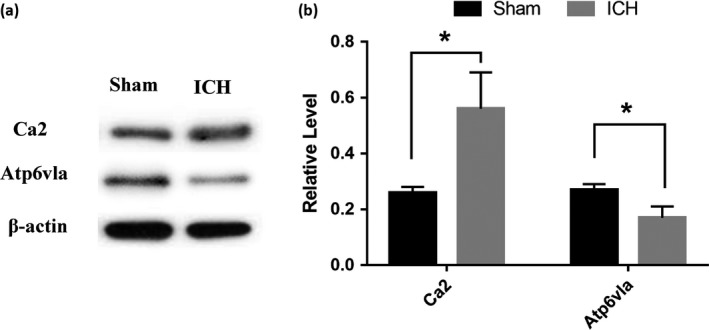
Validation of differential expression of Ca2 and Atp6vla identified by LC–MS/MS. A, WB analysis to verify selected differentially expressed proteins. A representative result of WB shows the expression levels of Ca2 and Atp6vla in the brain tissue of sham and ICH group. B, Ca2 was upregulated, and Atp6vla was downregulated in ICH group, which is consistent with the iTRAQ results. Error bars indicate *SD*; *p < 0.05 versus sham group; *n* = 5 per group

Based on the results of the bioinformatic analysis and the correlations with disease pathogenesis, two candidate DEPs, namely, Ca2 and Protein Atp6v1a, were selected for validation using western blotting. Compared with that of the sham group, the protein expression of Ca2 was notably higher in ICH rat brains (*p* < 0.05). In addition, the protein expression of Atp6vla was remarkably lower in ICH brains than in brains of the sham group (*p* < 0.05). Western blotting bands and their relative level calculations are shown in Figure 5. The results of western blotting tests were consistent with the iTRAQ results. The western blotting analysis of Ca2 and Atp6v1a confirmed the iTRAQ data.

## 4 DISCUSSIONS

In our study, we used the collagenase‐induced ICH model. This model is a classic animal model that is widely used in ICH studies and recognized by researchers (Lee et al., 2008; Wang, Schretter, Clarke, & Lee, 2015; Yabluchanskiy et al., 2012). This model also mimics the hematoma expansion of continuous bleeding that occurs naturally in ICH patients (Del Bigio, Yan, Buist, & Peeling, 1996). Subsequently, we screened 201 DEPs (75 downregulated and 126 upregulated) by iTRAQ‐based quantitative proteomics. In KEGG analysis, the pathway of nitrogen metabolism based on energy metabolism has been emphasized. Subsequently, the results of STRING analysis indicated that one of the top‐ranked networks of DEPs was tightly associated with energy metabolism. In addition, most proteins were enriched in energy metabolism in the classification of DEPs based on stroke pathophysiology. According to our data, the dysregulation of energy metabolism may be closely related to secondary ICH injury.

The mechanism of intracerebral hemorrhage is very complicated. The identification, classification, and analysis of DEPs should shed light on the molecular basis of ICH injury (Table 2). In this study, 85 significant DEPs were classified into 13 categories based on stroke pathophysiology. As shown in Figure 4, energy metabolism, inflammation and stress, structural proteins, coagulation cascade, and synapse‐related proteins ranked top five in the above classification. Numerous preclinical studies show that the interaction of OS and inflammation participates in the brain injury secondary to ICH (Aronowski & Zhao, 2011). This study reveals that alpha‐1‐macroglobulin (Pzp), serum albumin (Alb), apolipoprotein A‐I (Apoa1), and heat‐shock 27 kDa protein 1 (Hspb1) are involved in OS and inflammation. Several structural proteins including tubulin beta‐5, tubulin alpha‐1A, tubulin beta‐3 and tubulin beta‐4, neurofilament heavy polypeptide (Nefh), neurofilament medium polypeptide (Nefm), neurofilament light polypeptide (Nefl) and tubulin alpha‐4A chain (Tuba4a) are upregulated at 48‐hr after ICH in this study. The coagulation cascade can limit bleeding, while it can also trigger inflammatory cell infiltration, blood–brain barrier (BBB) damage and edema (Han et al., 2016; Sun, Keep, Hua, & Xi, 2016). We found that Ac1873 (Fga), T‐kininogen 2(Kng1), fibrinogen gamma chain (Fgg), fibrinogen beta chain (Fgb), and apolipoprotein H (Apoh) were implicated in the coagulation cascade. It is fascinating that synapses and synaptic plasticity have been associated with stroke recovery (Murphy & Corbett, 2009; Tamakoshi et al., 2014). This study shows that calcium/calmodulin‐dependent protein kinase type II subunit alpha (Camk2a), synapsin‐1 (Syn1), SH3, and multiple ankyrin repeat domain protein 1 (Shank1) are involved in synapse and synaptic plasticity. It should be noted that our data are consistent with many publications. However, there are few articles discussing energy metabolism in ICH. In addition, the core position of nitrogen metabolism pathway was found in the ICH brain for the first time. Therefore, the following discussions focus on energy metabolism.

### 4.1 Nitrogen metabolism

Nitrogen metabolism is a biological process of the nitrogen cycle and is closely associated with astrocyte‐neuron metabolism in the brain (Cooper, 2001; Mlody, Lorenz, Inak, & Prigione, 2016). Nitrogen in the brain is derived from the diffusion of ammonia or the metabolism of endogenous nitrogen‐containing substances (Cooper, 2001). However, ammonia lies at the core of the entire nitrogen metabolism pathway and is produced by amino acid metabolism and intestinal urease‐positive bacteria (Braissant, McLin, & Cudalbu, 2013). Ammonia metabolism is widespread in multiple organs of the human body. In the kidney, ammonia metabolism includes net ammoniagenesis and renal epithelial cell ammonia transport, inducing urinary ammonia excretion. The maintenance of sufficient renal ammonia metabolism may have a protective effect in patients with chronic kidney disease (Weiner & Verlander, 2017). In addition, ammonia was delivered to the liver, where it may be incorporated into urea or glutamine (Olde Damink, Jalan, & Dejong, 2009). The ammonia detoxification within the liver is highly efficient. Liver dysfunction and exercise act to provoke a change in ammonia homeostasis, which can influence neurological function (Wilkinson, Smeeton, & Watt, 2010). It is known that the toxic ramifications of ammonia primarily affect the brain and induce serious neurological impairment (Bobermin et al., 2015; Dasarathy et al., 2017). Increased ammonia (change in pH, electrolyte disturbances, membrane potential depolarization) is thought to lead to neurological dysfunction primarily by causing cellular swelling accompanied by brain edema and metabolic dysfunction (Bosoi & Rose, 2009; Dasarathy et al., 2017). Nevertheless, the role of nitrogen metabolism activation in ICH has not been reported. Nitrogen metabolism activation was first discovered, and 3 DEPs (Glul, Car1, and Ca2) were found to be involved in this pathway in this study. Nitrogen metabolism‐related ammonia toxicity may be a potential mechanism in the pathogenesis of ICH rats.

### 4.2 Glutamine Synthetase (Glul)

Glul is an ATP‐dependent enzyme and participates in the detoxification of incoming or endogenously generated ammonia (Cooper, 2011; Mlody et al., 2016). In the brain, Glul is predominantly located in astrocytes where it serves to maintain the glutamate–glutamine cycle, as well as nitrogen metabolism (Jayakumar & Norenberg, 2016). In the glutamate–glutamine cycle, glutamate taken up into astrocytes is not only converted into glutamine by the action of Glul, but also serves as an energy substrate resulting in ATP generation subsequent to metabolism of its carbon skeleton in the tricarboxylic acid (TCA) cycle (Parpura et al., 2017). In addition, Glul is indirectly involved in the glutamine cycle related to brain energy metabolism (Natesan, Mani, & Arumugam, 2016). Transient upregulation of Glul has been demonstrated in the hippocampal dentate gyrus during seizure acquisition in the amygdala kindling‐induced epilepsy model (Sun et al., 2013). A reduction in Glul activity has been revealed in the postmortem brain tissue collected from Alzheimer's disease patients (Hensley et al., 1995; Smith et al., 1991). Moreover, in patients with depression, a decrease in brain GLUL mRNA expression has been observed in specific regions (Choudary et al., 2005; Klempan et al., 2009; Sequeira et al., 2009). In addition, the upregulation of Glul has been shown to be neuroprotective in ischemic rats through ischemic postconditioning (Zhang et al., 2011). In this study, Glul was found to be upregulated, suggesting that enzyme‐based therapies may be an interesting and potentially important approach to combating glutamate excitotoxicity in ICH.

### 4.3 Carbonic anhydrases

Carbonic anhydrases are zinc enzymes that catalyze the interconversion between carbon dioxide and bicarbonate as well as other hydrolytic reactions (Supuran, 2016). In the brain, carbonic anhydrase is abundant and has been localized predominantly within the glia and choroid plexus (Chen & Chesler, 1992). The extracellular carbonic anhydrase has been revealed in the regulation or modulation of synaptic transmission via control of the extracellular pH shifts in the brain (Tong, Chen, & Chesler, 2006). In recent decades, carbonic anhydrase isoenzymes have become interesting therapeutic targets with potential inhibition or activation effects for the treatment of disorders such as epilepsy, idiopathic intracranial hypertension, and cancer (Supuran, 2015; Yildirim et al., 2015). Alternatively, Car1 and Ca2 are cytosolic isoenzymes (Guney et al., 2014). In this study, the upregulated of Car1 and Ca2 indicated that the carbonic anhydrase isoenzymes may exert a key effect on modulating neuronal signaling in ICH.

### 4.4 Network analysis

Network analysis revealed that the top‐ranked networks of DEPs were tightly associated with the coagulation cascade, energy metabolism, and inflammation. Moreover, the coagulation cascade and inflammation were investigated in previous studies. Therefore, energy metabolism was chosen for further analysis. In the PPI network, Ca2 was in the core position, while Atp6v1a, Atp6v0c, Atp6v0d1, Ppa2, and Atp6ap2 constituted a complete network. Ca2 has been discussed in nitrogen metabolism. These five proteins have been previously reported as being involved in energy metabolism. For these reasons, the Atp6v1a‐Atp6v0c‐Atp6v0d1‐Ppa2‐Atp6ap2 network may fine‐tune the energy metabolism of ICH.

### 4.5 Vacuolar H+–ATPases

Vacuolar H^+^–ATPases (V‐ATPases) are membrane‐associated, multisubunit protein complexes that function as ATP‐driven proton pumps (Toei, Saum, & Forgac, 2010) and include Atp6v1a, Atp6v0c, Atp6v0d1, and Atp6ap2. V‐ATPases use energy from the hydrolysis of ATP to ADP to pump protons across membranes and regulate pH in vesicular compartments, the cytoplasm and the extracellular space (Hinton, Bond, & Forgac, 2009; Zhao, Benlekbir, & Rubinstein, 2015). The acidity of intracellular compartments and the extracellular environment generated by V‐ATPases are crucial for diverse biological processes, including membrane trafficking, degradation of proteins, and proton‐coupled transport of small molecules (Forgac, 2007). In synaptic vesicles in neurons, the activity of V‐ATPases generates an H^+^ electrochemical gradient inside the storage organelles. This gradient provides the energy for driving the transport of all classical neurotransmitters into secretory vesicles (Edwards, 2007). In endothelial cells, the inhibition of v‐ATPase by concanamycin has been shown to hamper signaling pathways (Rac‐1, VEGFR2, Notch) that depend on vesicular recycling circuits, and, thus, represents an attractive novel and multifactorial approach for anti‐angiogenesis (Rath, Liebl, Furst, Vollmar, & Zahler, 2014).

Alternatively, Atp6v0c, which is the bafilomycin A1‐binding subunit of V‐ATPase, has a role in regulating the basal versus stress‐induced function of the autophagy‐lysosome pathway (ALP) and ALP‐associated substrate degradation in neuronal cells (Mangieri et al., 2014). Atp6v0d1, one isoform of subunit d in V‐ATPase, is critical in the embryonic development hypothesis, while gene disruption of Atp6v0d1 in mice caused embryonic lethality (Miura, Froelick, Marsh, Stark, & Palmiter, 2003; Wu, Xu, & Li, 2009). Atp6v1a, a catalytic subunit of V‐ATPase, is known to acidify lysosomes and to interact with the mechanistic target of rapamycin (mTOR) protein, which plays an important role in regulating autophagy (Kim et al., 2017). However, Atp6ap2 is called (pro)renin receptor (P)RR, which was originally discovered as a membrane sector‐associated protein of V‐ATPase. Atp6ap2 not only exerts enzymatic action but also causes angiotensin II‐independent intracellular signaling (Ichihara & Kinouchi, 2011). Taking these reports into account, we concluded that V‐ATPases (Atp6v1a, Atp6v0c, Atp6v0d1, and Atp6ap2) may participate in the pathophysiology of ICH by regulating the pH of intracellular organelles.

### 4.6 Pyrophosphatase (inorganic) 2

Pyrophosphatase (inorganic) 2 (Ppa2) is an energy‐linked enzyme and is located in the mitochondrial matrix (Lundin, Baltscheffsky, & Ronne, 1991). Ppa2 hydrolyzes inorganic pyrophosphate (PPi) into two phosphates, which is an essential activity for diverse biosynthetic reactions and for cellular energy metabolism (Guimier et al., 2016). Knockdown of Ppa2 has been shown to result in growth defects and loss of mitochondrial DNA in *S. cerevisiae* (Lundin et al., 1991), while Ppa2‐deficient cells might have limited ATP synthesis (Kennedy et al., 2016). In this study, Ppa2 was found to be downregulated suggesting that the failure of energy metabolism may be a fundamental mechanism in ICH.

Although this study revealed that the dysregulation of energy metabolism may be closely related to secondary ICH injury, this was limited to young rats. Therefore, the proteomic study of aged rats is the direction of our future research.

## 5 CONCLUSIONS

Our results suggested that NO‐based energy metabolism may be a potential pathophysiological mechanism in the secondary ICH injury. In addition, this study used iTRAQ‐based quantitative proteomics to unravel the hitherto unknown molecular mechanisms of ICH.

## CONFLICT OF INTEREST

The authors declared that they have no conflict of interests to this work.

## AUTHOR CONTRIBUTIONS

YW and TT designed the study and helped to coordinate support and funding. TL drafted the manuscript. TL, JZ, and PFL were responsible for the experimental operations. HJC and HGL performed the statistical analysis. TT and YW revised the paper. All authors read and approved the final manuscript.
